# Bridging theory and practice: a facilitation-driven game for reflective, collaborative veterinary education

**DOI:** 10.1186/s13620-025-00302-6

**Published:** 2025-10-17

**Authors:** Thomas-Julian O. Irabor, Clément Ngandjui Yonga, Makhan Danfakha, Jean-Luc Camille Hornick, Didier Gilbert Jean Marlier, Nicolas Antoine-Moussiaux

**Affiliations:** 1 Fundamental and Applied Research for Animals and Health, Faculty of Veterinary Medicine, University of Liège, Liège, Wallonie Belgium; 2 Institute for Research and Development, Campus UCAD/IRD de Hann, Route des Pères Maristes – BP 1386, Dakar, Senegal

**Keywords:** Interactive game, Veterinary education, Reflective learning, Clinical reasoning, Systems thinking, Biosecurity, Case-based learning

## Abstract

Veterinary education has long relied on lecture-based methods and conventional case-based learning, approaches that may not fully cultivate the critical and systems thinking skills required in today’s complex clinical environment. In response, we developed an innovative digital tool that integrates interactive gaming, narrative-driven expression, and structured debriefing into a cohesive simulation framework. Using backyard poultry management—a case selected for its regulatory and ethical complexities in European practice—as our testbed, the simulation engages students through a point-and-click game focused on laying hens. The tool presents a series of interactive decision points and intentional "traps" that prompt reflective discussion during post-game debriefing sessions.

A qualitative approach was employed to analyze data collected from focus group discussions, written group documents, and facilitator reflections during simulation sessions with veterinary students. Our thematic analysis identified three primary themes: (1) a multifactorial understanding of poultry health, (2) diverse perceptions of the veterinarian’s role in managing complex challenges, and (3) the simulation as an effective reflective trigger for enhancing clinical reasoning. These findings indicate that the simulation not only promotes active learning and systems thinking but also bridges the gap between theoretical knowledge and real-world clinical decision-making by linking clinical observations with broader regulatory, economic, and social considerations.

Despite limitations such as reliance on self-reported data and the absence of a control group, our study demonstrates that a facilitation-driven simulation framework can transform case-based learning into a dynamic, reflective inquiry process. This approach offers a promising alternative for enhancing educational outcomes in veterinary education and lays the groundwork for future research incorporating objective measures of competence and further refinements to balance structured guidance with learner autonomy.

**Supplementary Information:**

The online version contains supplementary material available at 10.1186/s13620-025-00302-6.

## Introduction

Veterinary education has long relied on lecture-based methods, but these approaches are increasingly seen as insufficient for developing the complex skills required in today’s multifaceted clinical environment [24]. To prepare future practitioners for the challenges they will face, it is essential to adopt more dynamic learning strategies that foster critical thinking and holistic problem solving.

One such strategy is case-based learning (CBL), an instructional approach that uses real or simulated scenarios—referred to as cases—to engage students in analyzing and solving context-specific problems. CBL encourages active learning by placing students in realistic situations where they must apply theoretical knowledge to make clinical decisions. However, despite its advantages, CBL can present challenges. Instructors sometimes resist its full adoption due to unfamiliarity with its methods, and students may find the open-ended, information-rich format overwhelming and daunting, which can hinder effective participation and deep discussion [19, 36, 39, 45].

Equally important is the concept of systems thinking. Systems thinking is defined as an approach to analysis that emphasizes understanding how various elements within a complex system interrelate, how feedback loops and dynamic interdependencies shape overall behavior, and how these components interact over time [1, 28, 32]. For veterinary professionals, this means being able to see beyond isolated symptoms to recognize the broader context—spanning medical, social, economic, regulatory, and environmental factors—that influences clinical outcomes [7, 8]. This approach is critical for developing effective, sustainable strategies in complex clinical scenarios.

Research shows that interactive games have the potential to enhance systems thinking skills by encouraging teamwork, fostering the sharing of diverse perspectives, and engaging learners in dynamic problem-solving exercises [6, 29, 42]. In parallel, narratives have been used effectively to capture and communicate the intangible aspects of complexity—such as intuition, purpose, and uncertainty—that are difficult to quantify but are vital for a deep understanding of complex systems [43]. However, many games that employ narratives focus primarily on engagement and motivation, but skip the guided reflection that turns those experiences into real, lasting learning [51].

To address challenges in case based learning, our study intentionally integrates three critical elements: interactive gaming, narrative-based expression, and structured debriefing into a single educational tool. We developed an online point-and-click game that employs a systemic, interactive narrative specifically designed to highlight complex interactions. The narrative, which provides the reader the opportunities to decide the direction of the story often at a key plot point [25], is also purposefully structured to withhold certain information, shape character roles, and incorporate visual cues that prompt specific mistakes or oversights. These elements serve as intentional"traps"that are triggered during in-depth debriefing sessions, where the instructor guides students through reflective analysis of their decision-making processes. This structured reflection is essential for experiential learning, as it enables learners to connect their gameplay experiences to broader clinical insights [29, 30].

We used this integrated approach as a case study in backyard poultry management—a context selected for its relevance to European practices and its inherent regulatory and ethical challenges [16, 23, 27, 37, 48, 50]. By combining interactive gaming, narrative-driven expression, and structured debriefing, our study demonstrates how a systemic narrative can address the limitations of case-based learning, facilitate a deeper exploration of complexity, and foster the reflective skills necessary for holistic clinical reasoning in veterinary education.

## Research objective

The objective of this paper is twofold. First, to investigate how future veterinarians interpret an interactive narrative on backyard poultry farming, emphasizing the veterinarian’s role and the interplay between evolving EU legislation, social trends, and the multifaceted challenges of poultry management. Second, to examine the game framework as a reflective tool that supports discussions and fosters systems thinking through semi-structured debriefing, thereby facilitating deeper engagement with complex subjects.

## Methodology

### Rationale for the tool

The game was developed in response to evolving challenges in veterinary education across Europe. Increasing restrictions on poultry farm visits, driven by biosecurity considerations, and the understandable hesitation of some farmers to allow access to their facilities—whether due to the sensitive nature of industrial systems or the informal nature of smaller operations—have made practical training more complex ([49], p. 32). Furthermore, the European Association of Establishments for Veterinary Education (EAEVE) emphasizes the importance of poultry-focused training, even as universities face growing constraints in financial and human resources. Combined with a desire to streamline time-intensive activities, these factors have underscored the need for innovative teaching methods, including digital approaches.

In this context, the game was designed to provide an alternative means for students to gain practical insights while addressing these constraints. It aims to foster systems thinking, integrate sustainability considerations, and strengthen essential skills such as differential diagnoses, clinical methodologies, and consultation techniques. By aligning with the realities of poultry management in Belgium, particularly in Wallonia, and incorporating zoonotic disease detection protocols, the game ensures its relevance and utility in preparing students for modern veterinary practice.

The connection between the game’s content and students’ career interests was of importance as well. At the Faculty of Veterinary Medicine here in Liège, Belgium, the majority of veterinary students are primarily interested in companion animals, with little to no focus on production animals, including poultry. Many students come from urban backgrounds, often with limited or no prior exposure to poultry farming. For some, basic interactions with chickens, such as handling them, are entirely new experiences. These factors create a disconnect between the students'existing interests and the perceived relevance of poultry management training. Currently, students receive a course on poultry diseases during the second semester of their fifth year, but attendance is low, and many show limited engagement with the subject. Over the years, the course has undergone significant restructuring, with theoretical lecture hours being progressively reduced-from approximately 18 hours in 2002 to just 6 hours today—further limiting the depth of exposure to poultry medicine.

To bridge this gap, it was critical to frame poultry management training in a way that connects with broader veterinary trends, such as the growing role of backyard poultry in urban and suburban settings [37]. These shifts have introduced poultry into small animal clinic caseloads, often as companion animals requiring care that blends production-focused practices with the expectations of emotionally invested owners. By aligning game narratives with these real-world developments, the goal was to help students recognize the relevance of challenging initial impressions about production animals even among those primarily focused on companion animal practice. This approach ensured that poultry management training was contextualized within the broader scope of modern veterinary practice.

### Design and development

The game was developed as an online interactive point-and-click experience using the Genialy® platform. Development of the game began with a foundational Word document that integrated screenshots of the emerging Genialy® interface, a scripted narrative, and spaces for stakeholder commentary. This format allowed expert reviewers—including two Belgian professors (specialists in Avian medicine and animal management/production) and two assistant professors (one in Avian epidemiological and biosecurity risk assessment, the other in educational game design for systems thinking)—to comment on narrative elements, propose revisions, and ensure that the evolving scenario met practical veterinary needs and complied with Wallonia (French speaking Belgium) regulations. The assistant professor focusing on educational game design adapted these inputs into the Genialy® platform, modifying game mechanics and interactive paths.

The backyard poultry context in this game consisted specifically of laying hens. Although the narrative may have some applicability to other poultry types, it concentrated on egg production and typical health challenges in laying hens rather than meat chickens or dual-purpose breeds. This design choice matched the educational objectives of understanding biosecurity, clinical reasoning, and regulatory requirements in a setting where social trends for smaller flocks for egg and meat production were common. The initial scenario plan featured 10,000 birds, but stakeholders recommended reducing that number to 250 to better represent the complexity of smaller-scale backyard operations. By limiting the flock to 250 chickens, the design highlighted the legal threshold for Belgian poultry farms exceeding 200 birds. A small, mobile coop also reflected conditions frequently found in local backyard setups.

Students navigated eight interactive pages that appeared to offer multiple entry points but ultimately followed a structured pathway. Simple instructions, pop-up windows, and highlighted prompts guided them to crucial details, although the effectiveness of these cues was not formally tested. Certain features remained hidden until activated by the user, managing the pace of information and preventing overload. Visual materials were generated via ChatGPT Pro’s DALLE tool, enabling prompt-based creation of coherent imagery and reducing the time spent sourcing photos.

### Scenario

On the first page, students received a brief introduction to the point-and-click experience and instructions to use a document for recording their observations, differential diagnoses, and recommendations throughout the game. On page 2, they assumed the role of Dr. Marie, a veterinarian at her clinic, as she responded to a call from Mr. Dupont about an illness in his backyard poultry flock.

On page 3, students were instructed to choose three out of six predetermined questions to build an initial clinical history with Mr. Dupont over the phone. These questions covered poultry clinical signs and symptoms, vaccination status, recent changes in the flock or environment, previous treatments, and the timeline of the illness. Although we intended to restrict the choice to three questions, technical limitations in the Genialy® platform resulted in all six options being visible. To ensure every student received essential information, we embedded a trigger on the question about the birds’ symptoms that, when selected, made the button to proceed to the next page appear. We prioritized this question because we believed it was fundamental to a proper clinical history, ensuring that all students could advance in the simulation.

On page 4, students were invited to select five of six items to carry for an onsite investigation at Mr. Dupont’s farm. These items ranged from protective gear (masks, gloves) to basic collection and note-taking tools (sample kits, paper/pen) and included a box of antibiotics. The inclusion of antibiotics served as an intentional choice point, prompting discussion on antimicrobial stewardship during debriefing.

**Fig. 1:**
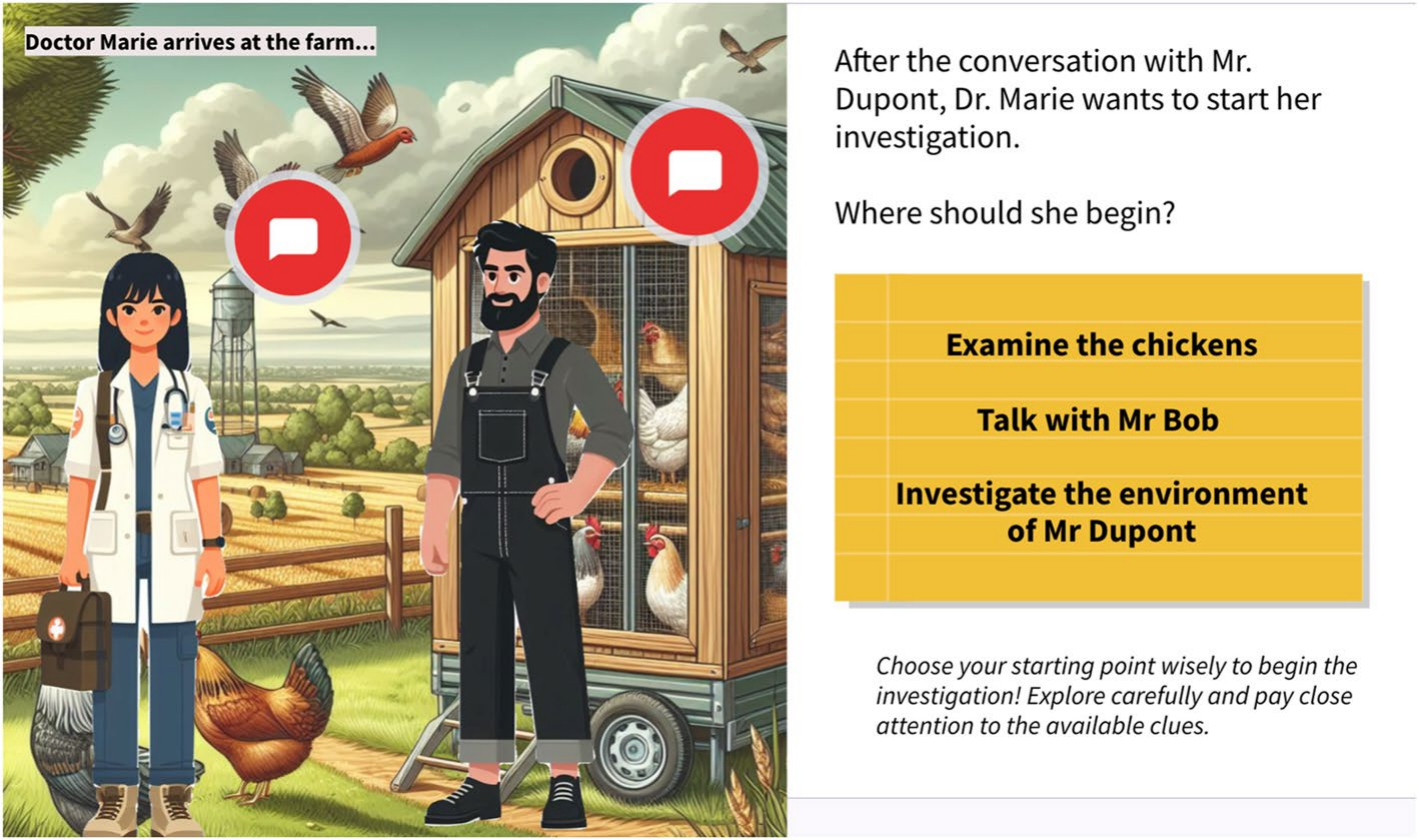
Slides from the avian game The game offers students’ multiple options to begin their investigation. On the right side of the screen, three starting locations are presented, giving students the freedom to choose where to begin their exploration

After finalizing their choices, students moved to page 5, where they traveled to the farm and selected where to begin their investigation (Fig. 1). They could choose to examine the flock on page 6, speak with Mr. Bob—the farmhand who also managed his own smaller flock, raising the possibility of cross-contamination—on page 7, or assess the environment on page 8. The game design intention emphasized starting with the environment, which provided key context for recognizing biosecurity risks and external risk factors. Students who began with the environment maintained access to both the flock and Mr. Bob, gaining a more complete understanding of the situation. In contrast, those who chose the flock or Mr. Bob first lost access to the environmental context, creating knowledge gaps that emerged later. To mitigate this, we placed Dr. Marie’s icon on various pages; clicking her icon allowed students to return to the farm and revisit pages they had missed due to an earlier choice.

The environment page, like other investigative areas, featured clearly visible elements with flashing cues that highlighted key information. Some details remained hidden, requiring students to actively search and apply their observational skills [11]. This escape game–inspired design added an extra challenge, as not all students uncovered the same details. The resulting disparities in knowledge became a focal point during debriefing, where students reflected on what they missed and how those gaps influenced their decision-making.

General observations noted clinical signs such as respiratory difficulty and bloody diarrhea, along with a 20% decrease in food and water consumption, a weekly drop in egg production of approximately 3%, and a mortality rate of 5% after two days. These clinical indicators, integrated into the narrative, were consistent with legislative criteria established by AFSCA—the Federal Agency for the Safety of the Food Chain, which safeguards the quality and safety of foodstuffs as well as plant, animal, and human health in Belgium. Under those rules, treatment cannot begin until Newcastle disease and Avian Influenza are both definitively confirmed, so every possible diagnosis in the game was viral [2]. Additionallly, conversations with Mr. Bob covered topics such as feeding and watering routines, wild-bird exposure, and the local distribution of eggs and meat.

### Facilitation-driven game design

We designed the game with debriefing in mind so that each key decision, character interaction, or missing piece of information naturally led to conversation during the post-game session. For instance, the narrative presented a suspected viral disease that required mandatory laboratory detection and reporting before treatment. This stage referred back to a moment when students chose five investigative tools out of six, including a box of antibiotics. Facilitators used the antibiotics option as a talking point to discuss stewardship, treatment rules, and legal obligations.

**Fig. 2:**
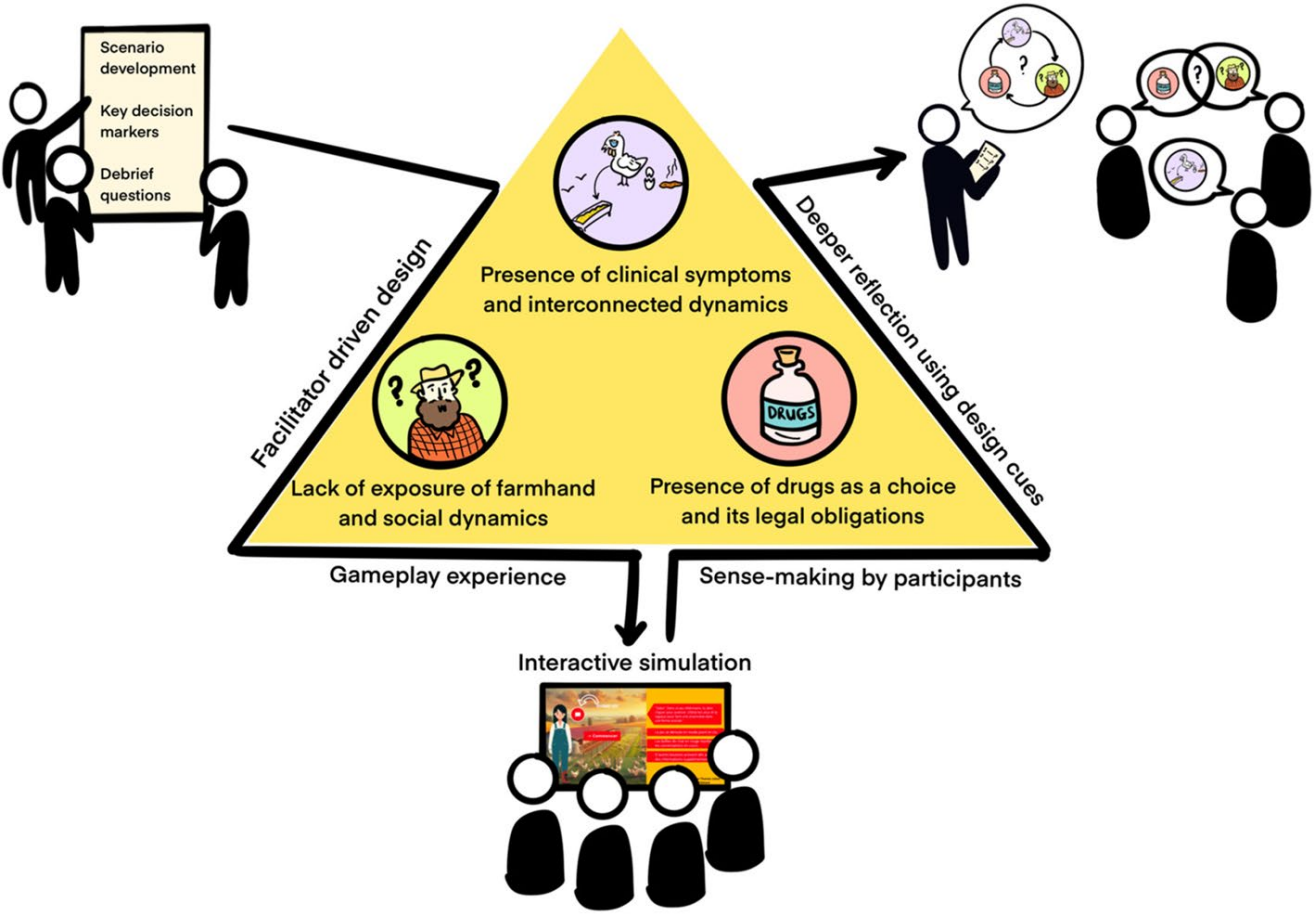
Facilitation-driven game design: This triangular diagram highlights the main pillars of the game design in the center: the presence of clinical symptoms and interconnected factors, the farmhand’s lack of knowledge and social dynamics, and the availability of medication (and its legal implications). Surrounding the triangle are small stick-figure groups illustrating how scenario development, decision markers, and deeper design cues guide the gameplay experience and shape participant understanding. The bottom of the figure shows the interactive narrative being played, while arrows flow toward post-game sense-making. This visual layout underscores how each component—decisions, social cues, and missing information—naturally leads into structured debriefing

Social cues played a major role as well. We depicted Mr. Bob, the farmhand, as uninformed to test how students filled knowledge gaps and handled wrong assumptions. His questions and claims encouraged players to correct misunderstandings, which then became part of the debriefing, as shown in Fig 2. Meanwhile, students who skipped checking the environment lost crucial clues, prompting further discussion on how missing data affected decision-making.

We structured a facilitation guide to provide a clear context for the study, obtain consent, and outline the research objectives. We organized it around eight key questions designed to cover a range of anticipated cues in the narrative, including the rationale behind the students’ clinical history, their choice to administer or withhold medication, therapeutic interventions, the necessity of environmental investigation, compliance with regulatory requirements, appropriate biosafety measures for a mobile poultry house, and reflections on the learning experience. This design ensured that discussions encompassed both the clinical approach and broader regulatory and ethical considerations. For a complete overview, please refer to the document in the Annex (Fig. 2).

### Learning activity

Between February 21 and May 15, 2024, Master’s students in veterinary medicine at the University of Liège, Belgium, enrolled in a mandatory poultry medicine and management course. Each Wednesday during this period, a different group of students took part in a single-day learning activity featuring the narrative-based game. The day before each session, those students watched videos of small-scale poultry farms to learn real-world contexts. Before starting the game, they attended a feedback meeting to share observations and review Belgian poultry regulations and policies. This preparation set the stage for the hands-on exercise. Completing the activity fulfilled that week’s course requirements, and although participation in the related research study was optional, all students volunteered. An assistant professor in avian medicine facilitated every session, providing the necessary subject-matter expertise.

**Fig. 3:**
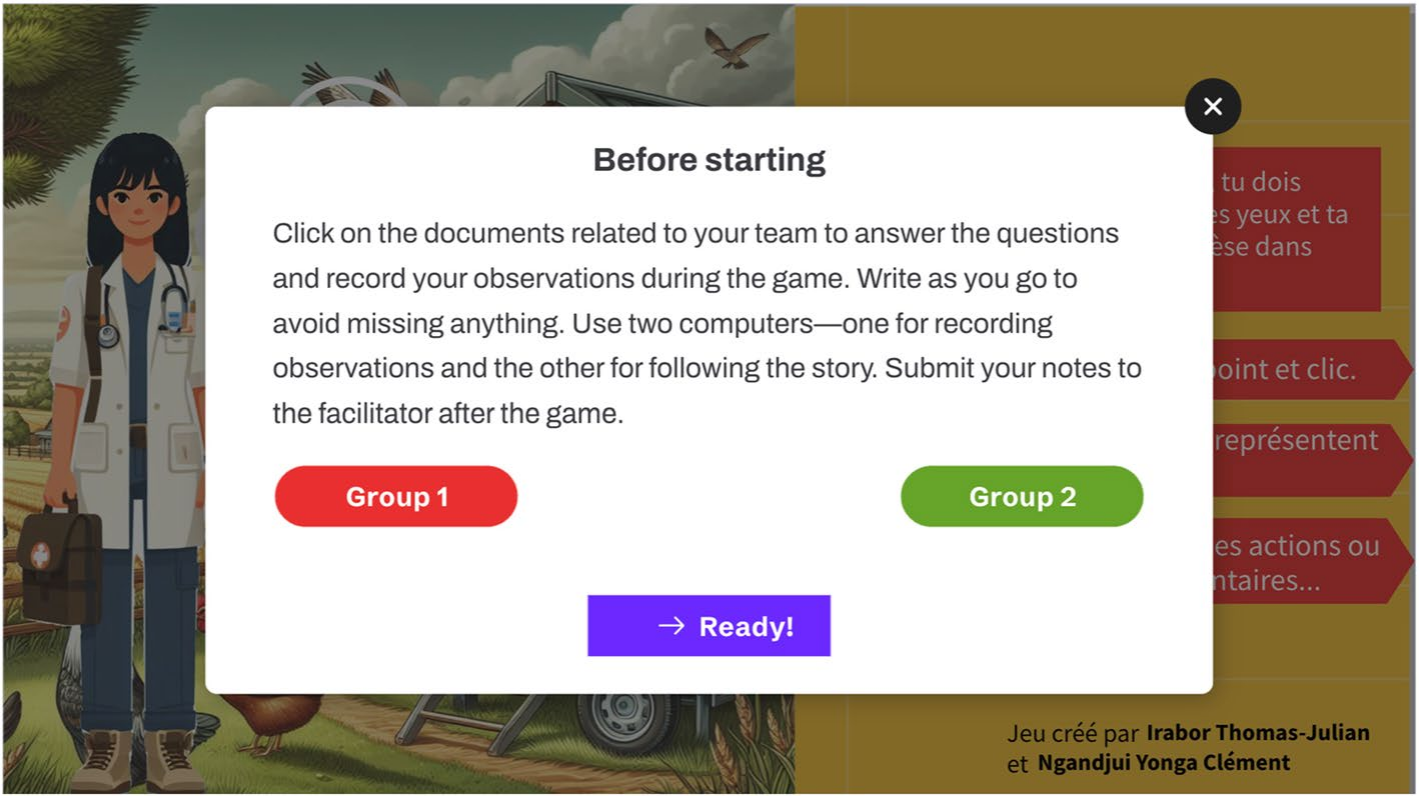
Illustration depicting how the document was integrated into the game: Prior to starting, participants accessed the document linked to their group by clicking on the provided link. They completed the questions in real-time during gameplay, ensuring their responses were aligned with the unfolding scenario

Additionally, an online document containing required response questions was integrated into the game, as shown in Fig. 3, and was accessible to the students via a provided link before the game commenced. This document, an online Google doc, served as a means for student groups to keep track of their advancements in the game and a way for the facilitator to compare their individual reflections and what emerged from the guided discussion.

The document begins with an identification section. Here, group details are listed, including last names, first names, and group numbers. The following sections consist of a series of clinical and observational questions related to the farm visit. The first question asks how the clinical approach was conducted at Mr. Dupont’s farm. Next, the document asks which potential health risks were noted during the exercise. Following the risk assessment, the document prompts for the potential symptoms observed during the game. A key part of the document is a table where the responder must correlate observed sanitary risks with the symptoms and suggest differential diagnoses. The table is structured into four columns: one for the sanitary risks, one for the symptoms, one for possible diagnoses, and one explaining the reasoning behind each diagnosis. After the table, there is a section asking which diagnostic samples should be collected to confirm the suspected diagnosis. This is concluded by a question asking for recommendations or advice to give to the farmer.

### Participants

Conducted within a classroom setting, each student group was required to bring at least two laptops or tablets. One for engaging with the game narrative and another for taking notes based on provided documents. Attendance averaged 10–13 students per session, who were split into two groups of 5–7 (78 students in total). Groups formed naturally based on where students sat and their own choice of whom to work with.

### Ethical consent

The university’s faculty ethics board granted an exemption for this study protocol (2024–152), and all participating students provided informed consent for audio recording and use of their written materials.

### Data collection

The study used three types of data: insights from focus group discussions, written group documents, and the facilitator’s reflections. At the end of each session, all students participated in a focus group discussion where they shared their experiences, explained their clinical approach, and reflected on how policies and regulations influenced their decisions. These discussions, conducted immediately after the game and written tasks, allowed for diverse perspectives while the facilitator guided conversations to identify strengths and areas for improvement in the students’ reasoning processes. To address challenges such as overlapping voices and background noise, a high-quality recording device was used to ensure clear data collection. Each group completed a written document during the game, which served as a baseline for the debriefing session. The students used this document to recall the decisions they had made throughout the simulation, facilitating a structured discussion during feedback. Additionally, the facilitator’s reflections provided insights into how his use of the design-led cues influenced the overall learning experience.

### Data analysis

We adopted a thematic analysis guided by Braun and Clarke’s framework [12, 13, 18]. By integrating data from focus group discussions, group documents, and facilitator reflections during the debriefing sessions, we ensured a systematic and transparent process that yielded both deductive (research-question-driven) and inductive (emergent) insights.

All audio recordings from the focus group discussions were transcribed in French to preserve participants’ exact wording. Two independent coders were involved: one closely familiar with the game’s design, and one external researcher. This setup enhanced quality control by mitigating potential biases from prior knowledge of the tool.

Each coder repeatedly read the transcripts, along with the written group documents. They took notes on prominent ideas or noteworthy excerpts—such as references to specific regulations, farm management practices, or reflections about the game design. This immersion helped identify preliminary patterns across the data.

The coders independently coded the transcripts line-by-line, annotating verbatim quotations with short descriptive labels (e.g., “biosecurity barriers,” “economic constraints,” “veterinarian as adviser”). An Excel sheet was used to store these codes, along with contextual details and any reflections on how the facilitator’s questioning might have influenced participant responses. Because the group documents primarily served the course’s instructional goals, they were only used for corroborating or clarifying points raised during the focus groups rather than as a stand-alone dataset for analysis.

After completing their independent coding, the two coders compared their code lists and discussed discrepancies. Codes that diverged were refined through dialogue, and duplicated codes were merged or re-labeled for clarity. This step yielded a shared coding framework that captured both recurring and unique ideas [35].

The agreed-upon codes were transferred to the Miro® platform, which enabled a visual mapping of relationships. Codes with conceptual similarities such as discussions of wild-bird contact, farmer responsibilities, or regulatory constraints were grouped into clusters. Miro’s drag-and-drop interface helped the team explore possible linkages and overlaps, providing an intuitive way to see how ideas interconnected.

Clusters were then examined to determine broader themes. Some clusters aligned readily with the study’s guiding questions (e.g., “Biosecurity and regulatory awareness”), while others surfaced inductively (e.g., distinct ways students perceived the veterinarian’s communication role). Themes were selected based on frequency (how often they recurred across transcripts) and uniqueness (novel insights that warranted deeper exploration) [34]. The process balanced these factors so that the most salient themes-those that consistently appeared across groups-were retained, while still incorporating new perspectives that enriched understanding.

Once potential themes were agreed upon, each was given a concise name and definition reflecting its central idea. All verbatim quotes, including a subset from the written group documents, were re-examined to ensure that each theme accurately captured participants’ intended meaning. The bilingual coder then translated selected extracts into English for reporting, ensuring fidelity to the original French phrasing.

The refined themes were re-organized into three overarching categories, each reflecting a key dimension of the research objectives: Interconnected understanding of poultry farming challenges, The perception of the veterinarian’s role in backyard practices, and The game as a tool for reflective learning.

These overarching themes synthesized both deductive coding aligned with the study’s focus (e.g., biosecurity, regulations, economic constraints) and inductive elements that emerged organically (e.g., unexpected attitudes toward smallholders, the significance of peer collaboration).

In practice, many “gaps” or incomplete references (e.g., students’ confusion about vaccination thresholds or when to report suspected diseases) served as useful debriefing prompts. These were intentional design features and points where the facilitator could clarify misconceptions. The result was a thematic structure that not only captured students’ knowledge and misunderstandings but also illuminated how the tool and subsequent debrief facilitated deeper learning.

The final three themes were chosen because they best reflected the view of poultry health as a multifactor Issue (Theme 1), the differences in how students envision the Veterinarian’s role under evolving sustainability demands (Theme 2), and the value of the tool as a reflective trigger for prompting wide-ranging reflections on clinical reasoning (Theme 3). Each theme included both strong insights from the participants and less explored or conflicting views that signaled important areas for future instruction.

## Results

### Viewing poultry health as a multifactor issue

Participants described poultry health as shaped by clinical, social, environmental, economic, and legal factors. Many initially focused on clinical signs-such as coughing or sudden mortality-but quickly connected these to external influences. One participant explained, “there are plenty of external elements to analyze in order to pinpoint potential issues,” indicating a shift beyond isolated symptoms. They recognized that free-roaming birds, contaminated water, and inadequate housing could worsen disease outcomes. They also acknowledged financial constraints faced by smaller flock owners, noting “not everyone can afford a decontamination unit” and adding that some might skip recommended vaccines if they consider them too costly.

Comparing large commercial operations to backyard flocks, participants noted that an outbreak in 50,000 chickens, as opposed to 250, carried different economic stakes. In one discussion, they recalled how a professor’s advice in an earlier course to treat aggressively for a large flock might not apply to a small hobby farm, showing awareness of context-specific trade-offs.

They linked consumer demand for humane, organic poultry to “more and more small-scale farms and people keeping chickens at home.” While this shift reflected public interest in food quality and animal welfare, it also created new challenges. One participant described the “mismatch between supply and demand,” observing that higher production costs often led consumers to raise their own birds. However, these broader factors did not always emerge spontaneously until the facilitator directed attention to legal requirements and financial barriers.

During conversations about vaccination thresholds, students initially focused on disease risk until the facilitator introduced farm size regulations. One participant then commented, “Those with 99 birds won’t vaccinate, but they can still spread the disease.” Similarly, when discussing biosecurity, they first framed the issue as farmer awareness. Only after further prompts did they recognize economic constraints, concluding, “It’s financial… they don’t want to change their practices as long as they can get away with it.” Although participants proved capable of seeing these external influences, they did not always prioritize them without facilitator guidance, suggesting that outside prompts helped broaden their understanding of poultry health.

### Differences in how students envision the Veterinarian’s role

Participants agreed that veterinarians served multiple functions but held contrasting ideas on when and how to apply these roles. They acknowledged that veterinarians had to go beyond “performing the medical act.” In suspected avian influenza or Newcastle disease, they recognized that “the law requires not treating,” which meant prioritizing containment and reporting. They also debated whether to carry and administer antibiotics. One student insisted, “we don’t treat without tests… especially in large farms,” reflecting awareness of antimicrobial stewardship, while others argued it would be “more dire to let animals die” than to adopt a “cannot really do harm” approach. These views highlighted how students balanced immediate animal health needs against long-term concerns such as drug resistance.

They strongly promoted preventive strategies and sustainable practices. Participants suggested keeping feed and water indoors, installing barriers or nets, and restricting human traffic between flocks. One said, “if I have my hens, I won’t visit the neighbor’s hens,” and another noted biosecurity improvements should be introduced “step by step because otherwise they won’t do it.” When the facilitator clarified that flocks under 100 birds were not obliged to vaccinate, students connected the potential risk of unvaccinated backyard poultry to other farms. They linked such decisions to economic concerns, noting “the vaccine is expensive” and might not be seen as “cost-effective” for small flocks. Through these conversations, participants showed that veterinarians had to balance animal health priorities, regulatory obligations, and farmer socio-economic perspectives, underscoring education, communication, and gradual implementation of biosecurity measures.

Attitudes toward farmers who overlooked biosecurity also varied. Several empathized with their limited finances or knowledge, pointing out that “in backyard farms, there is no biosecurity at all… They don’t necessarily know the right measures.” Others applied negative labels, calling the fictional farmhand Mr. Bob “really stupid” and blaming him for spreading infection. When the facilitator urged them to consider economic or informational constraints, participants realized that alienating owners could undermine cooperation, and that effective communication might matter as much as technical skill.

### The game-based tool as a reflective trigger

Students described the tool as “well-made, interactive, and realistic,” noting how it prompted them to consider farm conditions, clinical signs, and the farmer’s statements before reaching conclusions. One participant highlighted the freedom it offered: “It allowed us to choose where to start,” a feature they felt mirrored real-world decision-making. They valued that “each option could bring something useful,” reflecting an understanding that different actions can still lead to relevant insights. This particular aspect of the experience created an opportunity for students to"talk things out and even vote."The freedom of choice required students to debate their priorities and decide collectively on key decisions, such as which investigative paths to follow.

Many contrasted this style of active learning with more passive classroom approaches. One participant stated, “It pushes us to think and not just repeat what we learned in class,” emphasizing problem-solving over memorization. Another added, “It’s the first time we’ve practiced like this with poultry farming. It really allows us to think about a different method of learning.”

Despite these positives, participants raised concerns about the game’s design. A recurring issue was the lack of real consequences for incorrect or rushed decisions, which they felt weakened its resemblance to real-life challenges. One student said, “The problem is that there are no consequences for our choices… There’s no difference.” They noted that limited accountability made the tool less immersive. Others mentioned that flashing elements in the interface “biased” their decisions, as it drew attention to certain options. Students also described instances where they could bypass question limits or had to repeat sections, interrupting the experience. Students recognized that, in professional settings, time pressures and accountability demand more measured, evidence-based decisions. Even though the narrative did not perfectly capture the unpredictability of real-life practice, participants agreed that the debriefing session afterward solidified lessons on broader responsibilities and potential outcomes, ultimately deepening their grasp of veterinary work where one student observed, “Without the instructor’s questions, we might not have realized we were missing the official reporting step”.

## Discussion

Research in professional education shows that case‐based, interactive learning broadens clinical thinking by revealing the many factors that shape decision-making. When learners engage with systemic case narratives-stories that include clinical, economic, legal, and social elements-they better understand that outcomes result from a mix of influences. For example, work on clinical reasoning among veterinary learners demonstrates that only a holistic approach can address the many layers of real cases [21]. Similarly, research in patient safety among medical students finds that errors rarely stem from a single cause but emerge from a network of contributing factors [22].

Interactive activities such as games and e-learning modules further encourage this broader perspective. One study found that a case‐based interactive course not only improved factual knowledge but also enhanced medical students’ systems thinking. In a similar study, veterinary students who practiced solving clinical scenarios by considering herd-level, client, and policy issues were able to apply this wider approach to new situations [44]. These findings suggest that interactive methods can shift learners away from a narrow, symptom-focused view toward a deeper understanding of complex, interrelated influences.

Structured debriefing is another key element in this learning process. In simulation-based training, guided debrief sessions allow learners to review their decision-making, pinpoint errors, and connect theory with practice. Studies in veterinary education show that such debriefing enhances reflective learning and leads to notable improvements in performance under pressure [4, 40]. In one study, thorough debriefing in emergency simulations reduced students’ cognitive load and improved their overall performance [47].

Educational games and simulations also provide a setting to explore ethical challenges and encourage group discussion. These methods require learners to face realistic dilemmas that involve multiple stakeholders. Research on serious games in veterinary ethics indicates that this approach effectively prompts reflection on moral challenges and professional responsibilities [20]. However, some participants noted that the lack of real-life consequences in these games limits their authenticity.

The practical example of backyard poultry farming illustrates the varied and informal nature of such settings. In Belgium, for instance, small-scale producers who do not meet minimum flock size requirements are often exempt from registration and health monitoring, leaving many flocks at risk due to weak biosecurity, limited veterinary follow-up, and insufficient vaccination [5, 17, 26, 37]. This context led students to reflect on the difficulties of applying uniform biosecurity measures in smaller farms. They recognized that flexible, collaborative strategies are needed to address the unique challenges of informal backyard poultry farming [23, 31].

Our framework was designed to tackle these challenges by incorporating a facilitation-driven debrief that supports both instructors and learners through reflective dialogue. Embedded cues guide students to analyze decisions together, linking clinical observations with broader issues such as economic constraints and legal requirements. In this way, the tool acts as a boundary object that connects direct teaching with participatory learning, providing a common space for students and educators to engage with complex issues [3, 14]. This shared space helps move learners from passive reception to active discussion of challenges like biosecurity and ethics, supporting theories that stress the value of such collaborative tools [9, 10, 41]. Moreover, the design instills confidence in facilitators by linking anticipated learning outcomes to the participants’ experiences. This framework provides a solid starting point for facilitators while still allowing for exploratory discussion through a semi-structured approach.

Although the narrative was applied to backyard poultry farming, its design framework can be adapted to other case studies. By modifying content while keeping the structure, the framework addresses the common challenge of linking theory to practice, a task often burdened by heavy preparation demands [33, 45]. Through gradual scaffolding, students develop the skills to analyze complex interconnections in clinical cases, moving from a linear approach to one that values active engagement and group inquiry [45, 46].

Nonetheless, challenges remain in this design. Meadows [32] argues that a system’s structure fundamentally determines its behavior. In our tool, the organized structure guides users along a predetermined narrative path, promoting reflective responses and ensuring the achievement of key outcomes. However, this explicit organization also introduces limitations: while it provides clarity and consistency, it may restrict independent exploration and critical thought. This tension between guided clarity and autonomous inquiry highlights inherent design constraints. Furthermore, using shared tools to foster understanding requires a careful balance. This process is time-consuming and must avoid overly constricting the discussion [10, 15]. Although facilitators are essential in guiding these discussions, their influence may sometimes steer responses if dominant voices prevail, underscoring the necessity for skilled moderation [38].

Beyond these design challenges, our study is limited by its reliance on self-reported student data, which might not capture the full complexity of clinical practice. The simplified nature of the game also raises questions about its ability to fully prepare learners for real-world systems, especially given the noted absence of real-life consequences. Without a control group or comparisons with other methods, it is difficult to attribute outcomes solely to the intervention. Despite these limitations, our study offers a flexible design framework that, with further refinement to balance guidance and independence, shows promise for enhancing case-based learning in various settings.

## Conclusion

Our study shows that integrating a facilitation-driven debrief design within an adaptable framework can effectively bridge theoretical knowledge with real-world clinical decision-making. Reflective cues embedded in the narrative prompt collaborative dialogue, allowing students to engage actively with complex issues such as biosecurity, economic constraints, and legal requirements. Although applied within the context of backyard poultry farming for strong course linkage, the design framework is versatile enough to be adapted to various case studies, addressing challenges like the heavy workload of case preparation and the difficulty of linking theory to practice.

This approach is important because it transforms case-based learning from a linear, passive experience into a dynamic process of reflective inquiry and interdisciplinary engagement. By serving as a boundary object, the tool creates a shared space where students and educators can integrate diverse perspectives, fostering a deeper understanding of multifactorial challenges in veterinary practice. This shift has the potential to enhance learning outcomes, encourage critical thinking, and collaborative problem-solving, which are essential skills in modern clinical settings. Looking ahead, our framework invites further exploration. While promising, the design’s explicit cues sometimes constrained independent exploration, and the reliance on self-reported data limits our ability to assess long-term impacts. Future research should work to balance guidance with learner autonomy, incorporate objective measures of competence, and compare outcomes with other teaching methods. Additionally, identifying the competencies required for effective facilitation with this framework will increase effectiveness and maximize its educational benefits. Such refinements will help ensure that this adaptable design framework can continue to evolve and meet the diverse needs of veterinary education in various contexts.

## Supplementary Information

Supplementary Material 1: Annex 1. Facilitators guide.

## Data Availability

No datasets were generated or analysed during the current study.
