# Supplementary material for: Bridging theory and practice: a facilitation-driven game for reflective, collaborative veterinary education
Source: Ir Vet J. 2025 Oct 17;78:24. doi: 10.1186/s13620-025-00302-6 (PMC12535145; doi:10.1186/s13620-025-00302-6)
Supplement: Supplementary file 1 — Supplementary Material 1: Annex 1. Facilitators guide. [file 13620_2025_302_MOESM1_ESM.docx]

**Annex 1**

**Facilitators guide**

We are currently testing a game designed to strengthen students' skills in the clinical approach to poultry health as part of a broader research study. This study investigates the use of a game, focusing on how interactive tools can enhance clinical reasoning and decision-making. As part of the study, we are collecting recordings during the debriefing session to analyze student responses and their application of clinical principles in simulated scenarios. Your participation, including consent to be recorded, is crucial for understanding the effectiveness of this teaching approach and for refining future educational strategies. Do you consent to being recorded?

**Questions**

1. What were the reasons for choosing the three specific questions during the clinical history phase?
   1. What other questions might you ask?
2. Why did you decide to take or leave the medications?
3. Considering there was a 20% decrease in food and water consumption, a weekly drop in egg production of about 3%, and a mortality rate of 5% after two days, would a therapeutic intervention be advisable? Why?
4. Based on your observations, what are the advantages of investigating Mr. Dupont's farm environment ?
5. In Belgium, from an institutional standpoint, what does the regulation prohibit in Mr. Bob's small-scale poultry farming (in terms of feeding and watering, in terms of sharing of eggs and meat)? Why?
6. What biosafety measure should be considered for a mobile poultry house? Why?
7. What lessons did you learn from this game (clinical approach)?
8. What are your impressions and suggestions?
